# Human single-chain antibodies that neutralize *Pseudomonas aeruginosa-*exotoxin A-mediated cellular apoptosis

**DOI:** 10.1038/s41598-019-51089-w

**Published:** 2019-10-17

**Authors:** Sirijan Santajit, Watee Seesuay, Kodchakorn Mahasongkram, Nitat Sookrung, Sumate Ampawong, Onrapak Reamtong, Pornphan Diraphat, Wanpen Chaicumpa, Nitaya Indrawattana

**Affiliations:** 1 Department of Microbiology and Immunology, Faculty of Tropical Medicine, Mahidol University, Bangkok, Thailand; 2 Center of Research Excellence on Therapeutic Proteins and Antibody Engineering, Department of Parasitology, Faculty of Medicine Siriraj Hospital, Mahidol University, Bangkok, Thailand; 3 Biomedical Research Incubator Unit, Department of Research, Faculty of Medicine Siriraj Hospital, Mahidol University, Bangkok, Thailand; 4 Department of Tropical Pathology, Faculty of Tropical Medicine, Mahidol University, Bangkok, Thailand; 5 Department of Tropical Molecular Biology and Genetics, Faculty of Tropical Medicine, Mahidol University, Bangkok, Thailand; 6 Department of Microbiology, Faculty of Public Health, Mahidol University, Bangkok, Thailand

**Keywords:** Bacterial toxins, Medical research

## Abstract

Targeting bacterial virulence factors directly provides a new paradigm for the intervention and treatment of bacterial diseases. *Pseudomonas aeruginosa* produces a myriad of virulence factors to cause fatal diseases in humans. In this study, human single-chain antibodies (HuscFvs) that bound to *P. aeruginosa* exotoxin A (ETA) were generated by phage display technology using recombinant ETA, ETA-subdomains and the synthetic peptide of the ETA-catalytic site as baits for selecting ETA-bound-phages from the human-scFv phage display library. ETA-bound HuscFvs derived from three phage-transfected *E. coli* clones neutralized the ETA-induced mammalian cell apoptosis. Computerized simulation demonstrated that these HuscFvs used several residues in their complementarity-determining regions (CDRs) to form contact interfaces with the critical residues in ETA-catalytic domain essential for ADP-ribosylation of eukaryotic elongation factor 2, which should consequently rescue ETA-exposed-cells from apoptosis. The HuscFv-treated ETA-exposed cells also showed decremented apoptosis-related genes, i.e., *cas3* and *p53*. The effective HuscFvs have high potential for future evaluation in animal models and clinical trials as a safe, novel remedy for the amelioration of exotoxin A-mediated pathogenesis. HuscFvs may be used either singly or in combination with the HuscFv cognates that target other *P. aeruginosa* virulence factors as an alternative therapeutic regime for difficult-to-treat infections.

## Introduction

*Pseudomonas* exotoxin A (ETA) is one of the most potent bacterial virulence factors produced by *Pseudomonas aeruginosa*, an opportunistic Gram-negative bacterium, which belongs to the clique of difficult-to-treat multi-drug-resistant ESKAPE pathogens. *P. aeruginosa* is the common cause of life-threatening nosocomial infections, endowing a new paradigm to the pathogenesis, transmission, and drug resistance of infections worldwide^1^. Infections caused by this ubiquitous pathogen can occur in any part of the body, causing otitis media folliculitis (hot-tub folliculitis), otitis externa (swimmer’s ear), keratitis (corneal infection), bacteremia, endocarditis, pneumonia, urosepsis, etc.^2–9^. Infections may be fatal for individuals who are already very ill, such as those in intensive-care units, particularly ventilator-dependent subjects and patients with cystic fibrosis, cancer, diabetes, trauma, surgery, as well as neonatal infants^10–12^. *P. aeruginosa* causes disease by using numerous virulence elements, such as enzymes (elastase, proteases), pyocyanin, cytotoxins and biofilm^13–15^.

ETA is an NAD^+^-diphthamide ADP-ribosyl transferase (EC 2.4.2.36). This toxin catalyzes the transfer of ADP-ribose moiety from NAD^+^ to the diphthamide residue (a post-translationally modified histidine residue) on eukaryotic elongation factor-2 (eEF-2) through covalent attachment. This reaction results in the termination of protein synthesis and eventually leads to cell death^16,17^. ETA is a heat-labile, 613-amino-acid protein (66-kDa) which is released to the extracellular environment^18^. It is the most intoxicating virulence factor of *P. aeruginosa*, which is remarkably toxic to mammalian cells with a single toxin molecule^19^. It is extremely lethal, i.e., possessing an LD_50_ of 0.2 μg per mouse upon intraperitoneal injection^20^. The toxin molecule comprises three distinct domains, i.e., receptor-binding domain or ETA domain-1A (residues 1–252), translocation domain or ETA domain-2 (residues 253–384), and catalytic domain or ETA domain-3 (residues 385–613)^21^. ETA domain-1A (ETA-1A) binds its cognate receptor, called the heavy chain of low-density lipoprotein receptor-related protein/alpha 2-macroglobulin, on eukaryotic cells; then the toxin-receptor complex internalizes via clathrin-dependent endocytosis. In the early endosome, the toxin is exposed to an acidic environment and consequently cleaved between R279 and G280 within domain-2 by the host furin protease^22,23^. The cleaved-off-C-terminal (37-kDa) portion exits into the cytoplasm and is transported via the Golgi apparatus to the endoplasmic reticulum (ER). C-terminal KDEL of the enzymatically active 37-kDa fragment binds to the protein retention receptor-1 (KDELR1) on the ER membrane, and is subsequently translocated back to cytosol where it inhibits protein synthesis by catalyzing the transfer of the ADP-ribosyl moiety of the oxidized NAD onto eEF-2^24–26^. The catalytic 37-kDa fragment and also the full-length-ETA (ETA-FL) have been shown to induce cellular apoptosis by causing depolarization of the mitochondrial membrane resulting in cytochrome c release; activation of caspases- 9 and 3; and inactivation of DNA repair enzyme [poly(ADP-ribose) polymerase (PARP)] in several physiological events, including chromatin de-condensation, DNA replication and repair, gene expression (e.g., *p53*, *cas3*, *cdc2*, *cyclin-B*, and *bcl-2*) and cellular differentiation^27–32^.

Besides targeting the bacteria by using the traditional anti-bacterial drugs, an alternative therapeutic strategy is targeting bacterial virulence factors pivotal for pathogenesis in the host. The latter approach provides many benefits, such as maintaining the host endogenous microbiome and creating less selective pressure to the bacteria *per se*, which potentially reduces resistance^33^. At present, no novel antimicrobials active against bacteria already resistant to most or all currently available anti-bacterial drugs are under advanced development. Thus, there is an urgent need for a broadly effective agent that can cope with multi-drug-resistant (MDR) pathogens. In this study, engineered fully human single-chain antibody variable fragments (HuscFvs) specific to *P. aeruginosa* ETA were produced *in vitro*, using phage display technology. The HuscFvs effectively neutralized ETA-mediated mammalian cell apoptosis. It is envisaged that the antibody fragments may be used either singly or in combination with other anti-bacterial agent(s) as a novel remedy/protocol for fighting drug-resistant pathogens. The strategy reported herein may be applied to inventing prototypic therapeutics for other members of the ESKAPE group, and other pathogens.

## Results and Discussion

### Recombinant ETA domain-1A (ETA-1A), domain-3 (ETA-3), and full-length-ETA (ETA-FL)

**Figure 1:**
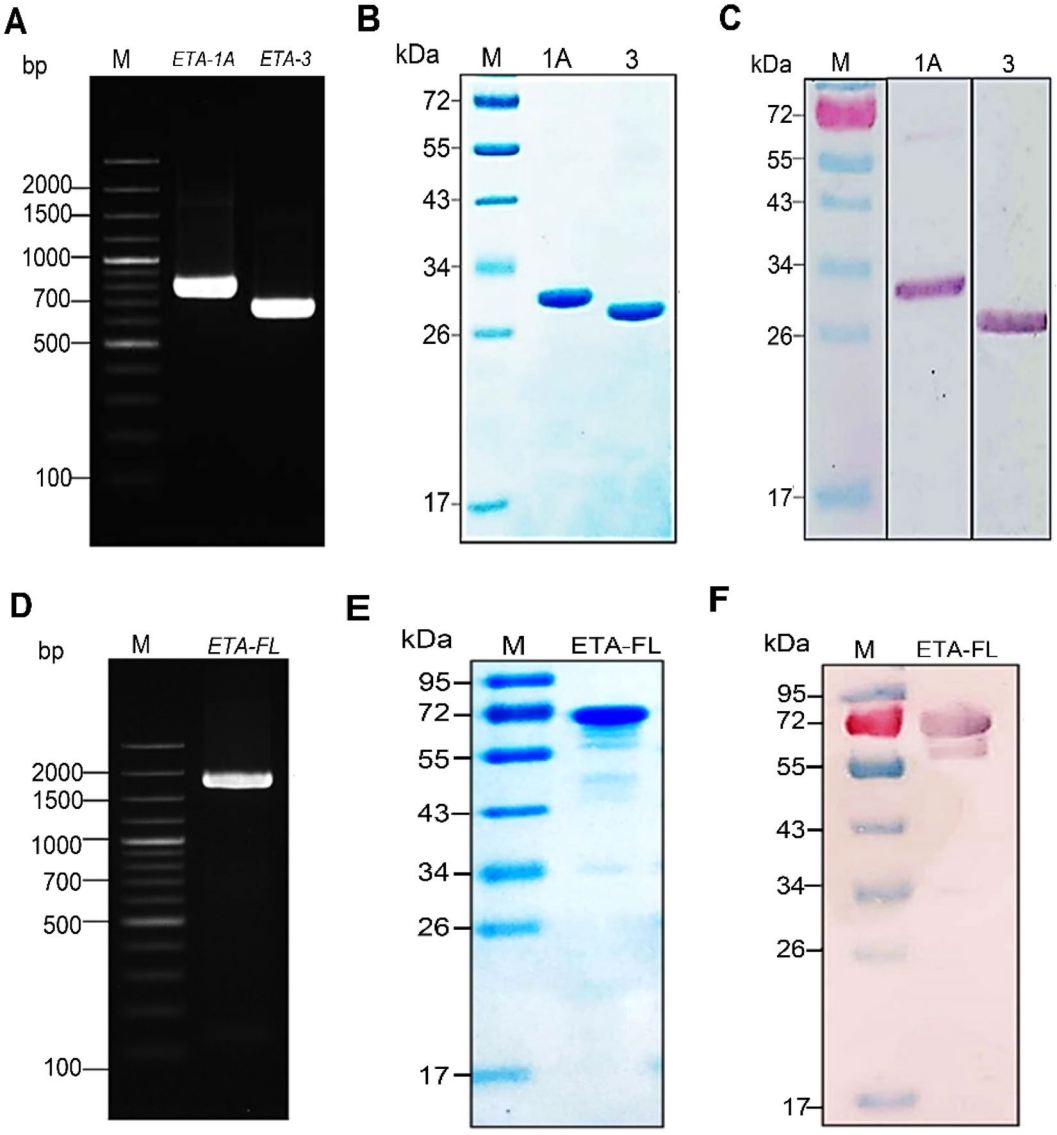
Production of recombinant ETA-1A, ETA-3 and ETA-FL. Panel A, amplicons of *ETA-1A* (756 bp) and *ETA-3* (627 bp). Panel B, stained SDS-PAGE-separated rETA-1A (28 kDa) and rETA-3 (26 kDa). Panel C, Western blot patterns of rETA-1A and rETA-3. Panel D, amplicon of *ETA-FL* (1,839 bp). Panels E and F, stained SDS-PAGE-separated rETA-FL (66.7 kDa) and the Western blot pattern of rETA-FL, respectively. Numbers at the left of panels A and C, DNA molecular size marker in base pairs (bp). Numbers at the left of Panels B, C, E and F are protein molecular masses in kDa. Full-length blots/gels are presented in Supplementary Fig. S1.

**Figure 2:**
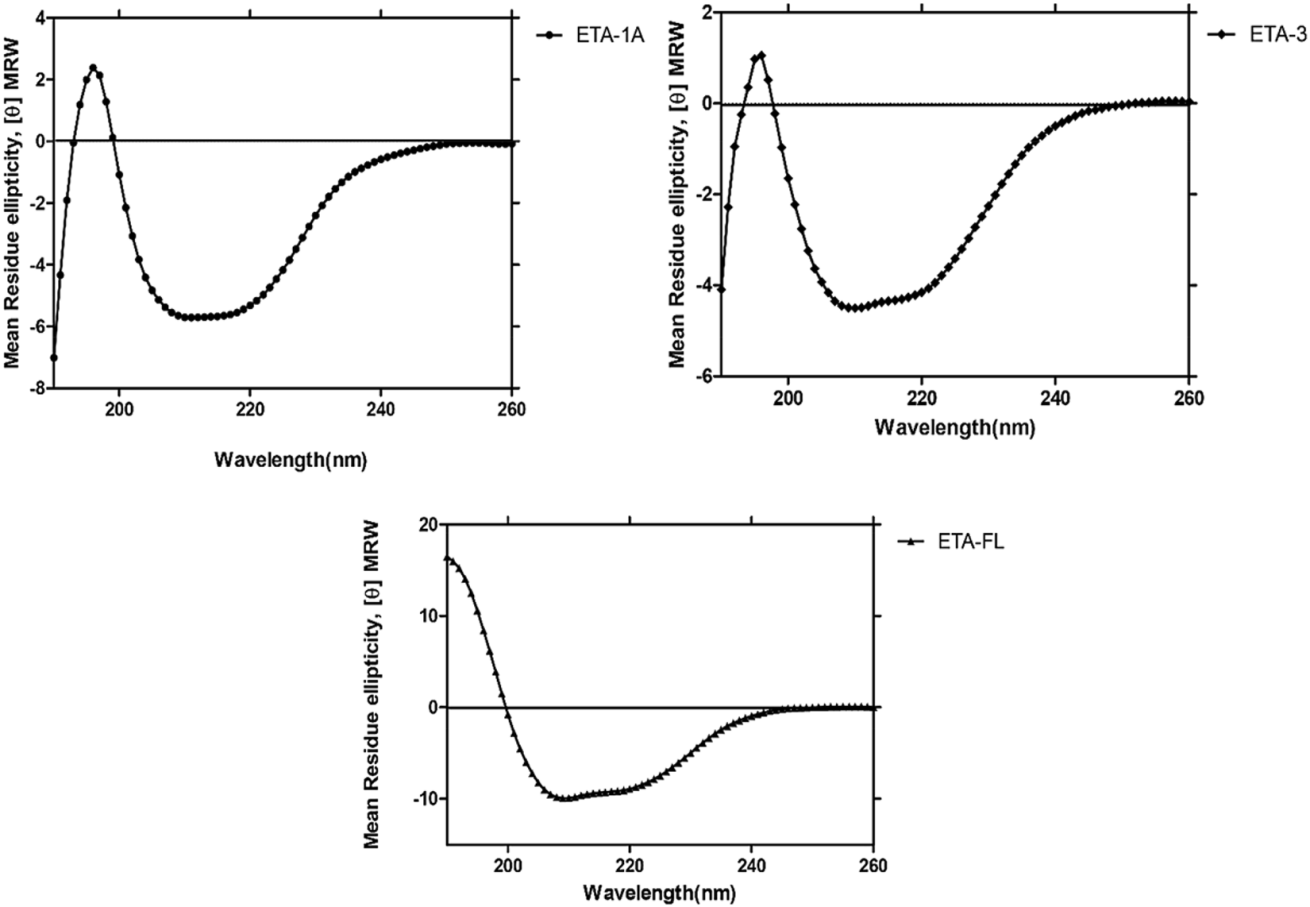
CD spectra in the far-UV region of the refolded rETA-1A, rETA-3, and rETA-FL.

Amplicons of *ETA-1A* and *ETA-3* are shown in Fig. 1A; the *ETA-FL* amplicon is shown in Fig. 1D. The *E. coli* clones carrying recombinant plasmids with the *ETA-1A*, *ETA-3* and *ETA-FL* inserts were grown under 1 mM IPTG induction; the recombinant proteins were successfully expressed. After purification and refolding, the recombinant protein of each preparation revealed only one protein band by SDS-PAGE and Coomassie Brilliant Blue G-250 (CBB) staining and Western blot analysis, with molecular weights of approximately 28, 26, and 66.7 kDa, respectively (Fig. 1B,C,E,F). LC-MS/MS verified that the purified recombinant proteins were *P. aeruginosa* proteins (Supplementary Table S1). The recombinant proteins were further characterized using far-UV Circular Dichroism (CD) measurements. They were found to acquire a predominantly alpha-helical structure (Fig. 2), which is characteristic of the ETA protein; the protein structures conformed to RCSB Protein database, 3B82^34,35^.

### Biological activities of recombinant ETA-FL

**Figure 3:**
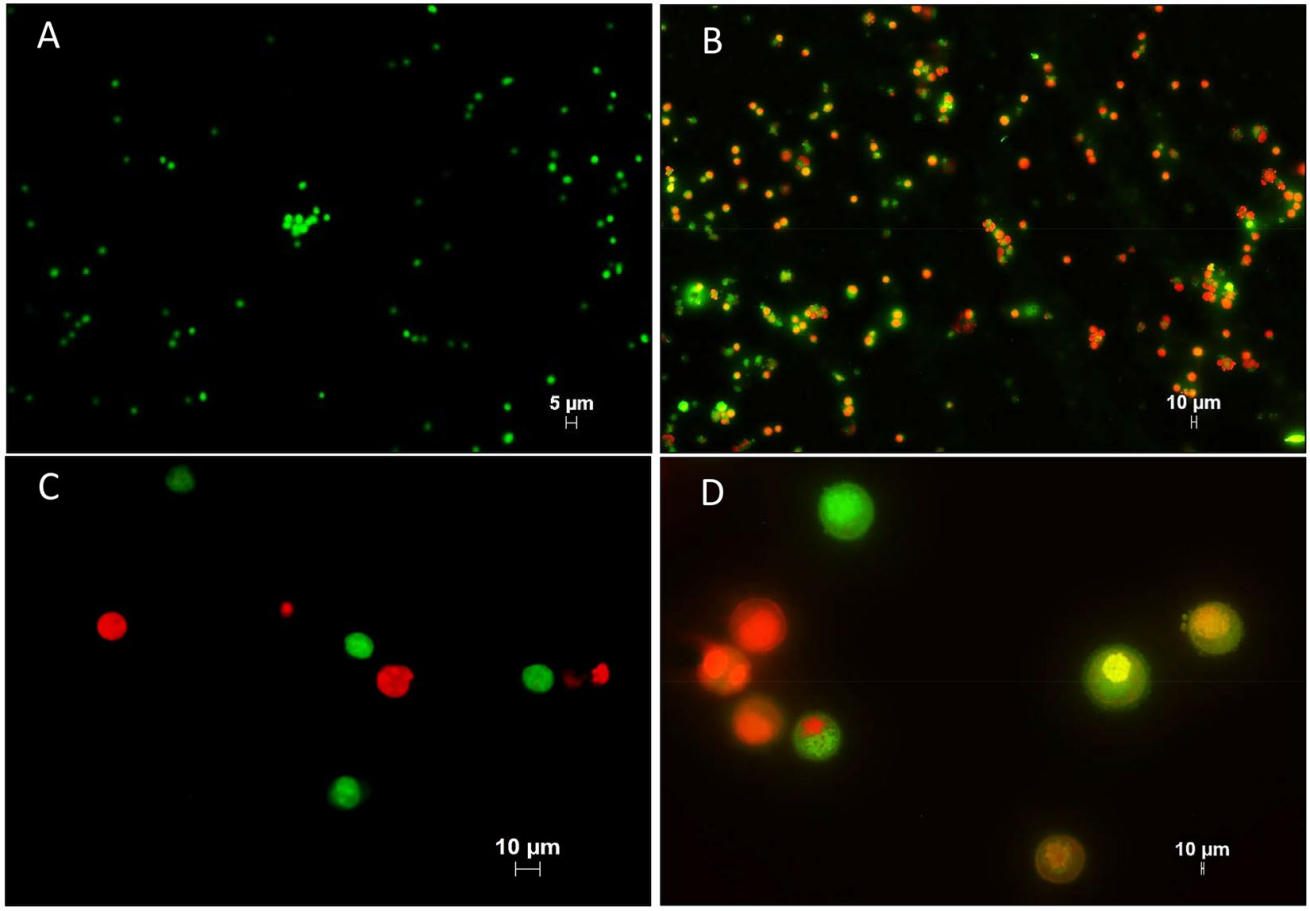
ETA-exposed-HeLa cells stained with AO/EB. Panel A shows normal HeLa cells without prominent apoptosis (10× magnification). Panels B–D, are HeLa cells treated with 3 nM of rETA-FL for 24  h at 10×, 20×, and 40× magnifications, respectively.

**Figure 4:**
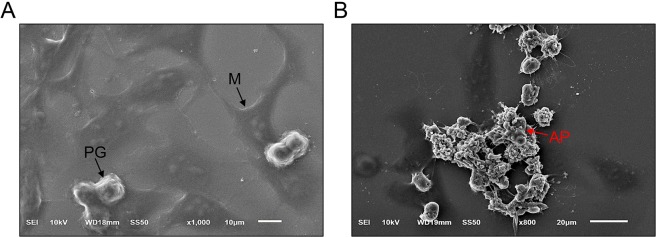
Scanning electron microscopic appearance of HeLa cells. Panel A, normal cells; the typical mature form without granule (M), and the progenitor form (PG) consisting of small and rounded cells with a smooth regular membrane. Panel B, after exposure to 3 nM of recombinant full-length ETA for 24 h; the apoptotic cell (AP) appears as a shrunken cell with numerous vacuolated membranes.

**Figure 5:**
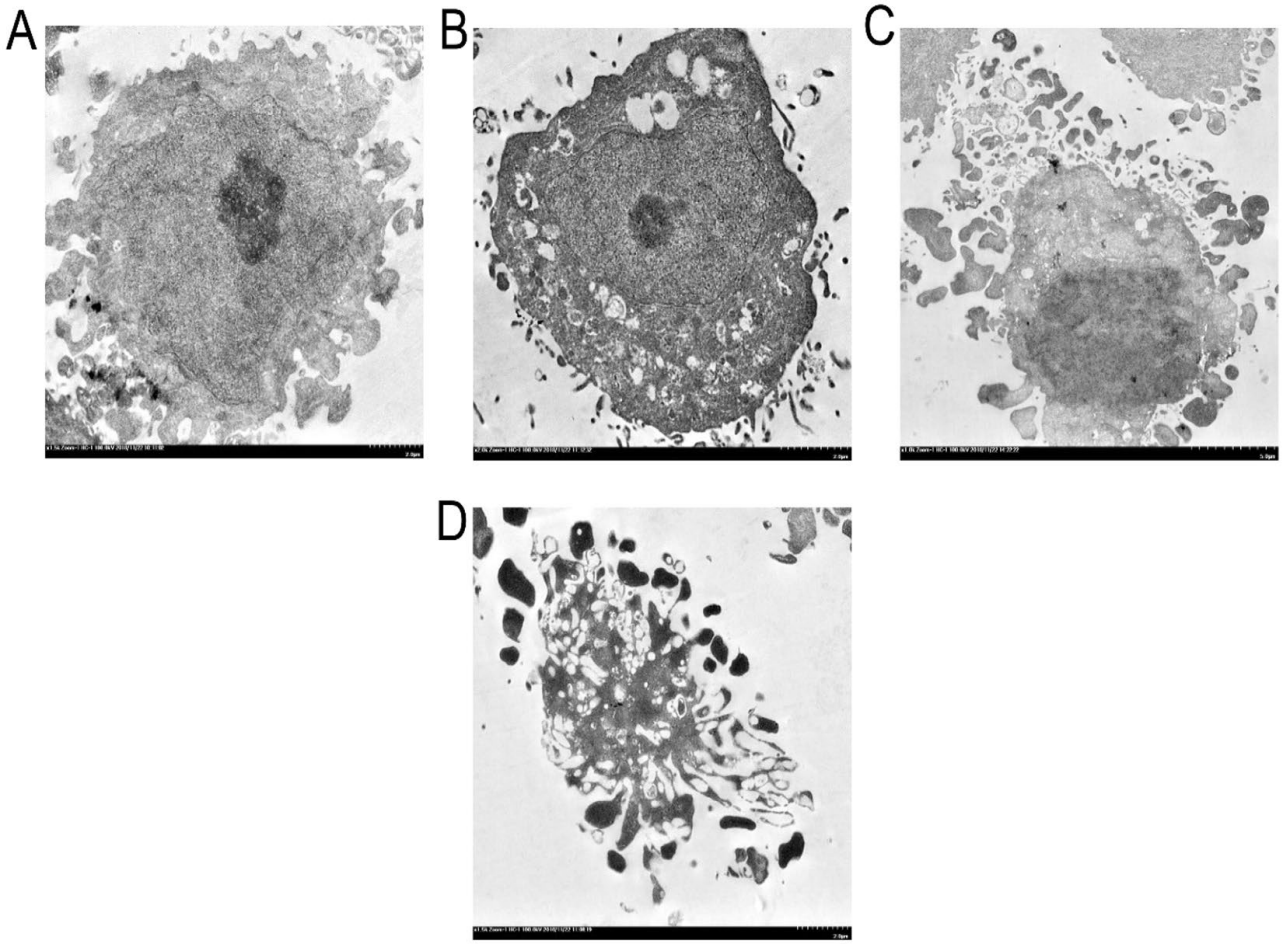
Transmission electron microscopic appearance of HeLa cells exposed to 3 nM of rETA-FL for 24 h. (**A**) Normal cell; (**B**) early apoptotic cell.

**Figure 6:**
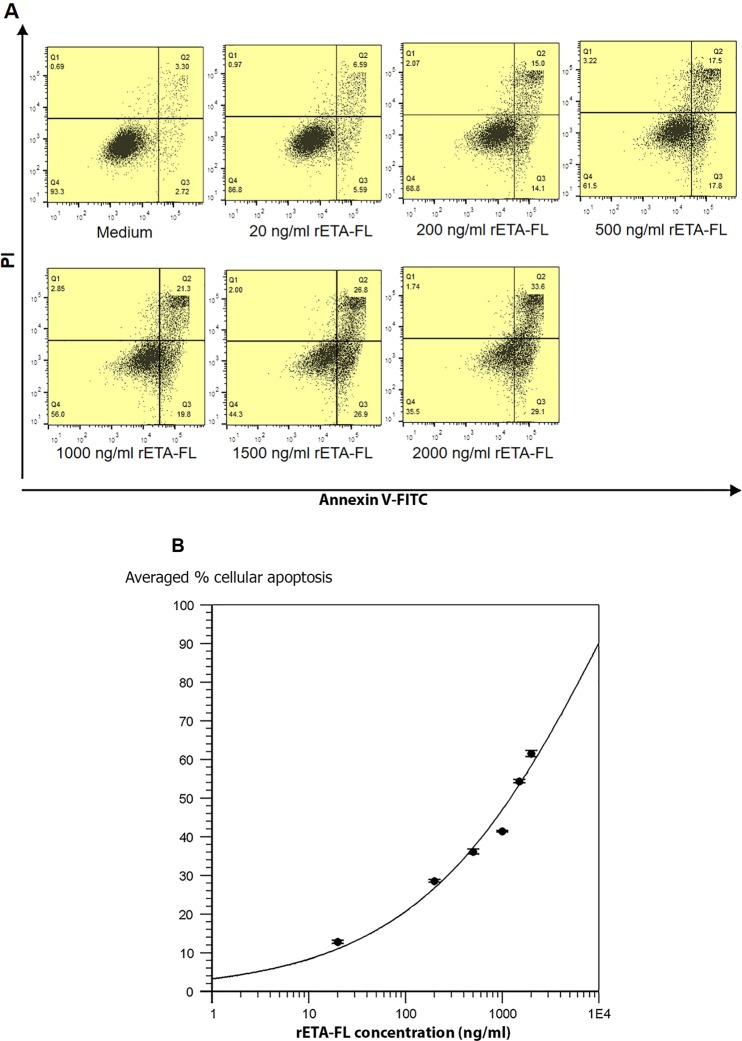
Cytotoxic effect of rETA-FL on HeLa cells revealed by staining the ETA-exposed cells with Annexin V/PI stains and flow cytometric analysis. (**A**) Percentages of Annexin V-FITC + PI+ in the HeLa cells treated with various concentrations of rETA-FL for 24 h. (**B**) Dose-response curve of rETA-FL on HeLa cell apoptosis.

The effect of recombinant ETA-FL (rETA-FL) on mammalian (HeLa) cells were determined using dual acridine orange/ethidium bromide (AO/EB) fluorescent staining, flow cytometric analysis, and ultrastructural studies by means of scanning electron microscopy (SEM) and transmission electron microscopy (TEM). The results revealed that the rETA-FL induced apoptosis of the HeLa cells was characterized by morphological changes and staining patterns, i.e., the early apoptotic cells showed green fluorescence with nuclear fragmentation or condensed chromatin, cellular blebbing and cytoplasmic vacuolization, while the late apoptotic cells exhibited orange-red fluorescence with condensed chromatin (Fig. 3). The ultrastructural study results by SEM and TEM for the ETA-treated cells are shown in Figs 4 and 5, respectively. The percentages of apoptotic cells after treatment with various amounts of ETA, determined by the Annexin V/PI staining and flow cytometric analysis, are shown in Fig. 6A. The average percentage of apoptotic cells increased in an ETA-dose-dependent manner, i.e., 12.79 ± 0.86, 28.55 ± 0.78, 36.15 ± 1.20, 41.4 ± 0.42, 54.35 ± 0.92, and 61.5 ± 1.70% for 20, 200, 500, 1,000, 1,500, and 2,000 ng ETA/ml, respectively. The background apoptotic HeLa cells in the medium alone was 6.02%. The minimum rETA-FL concentration that induced apoptosis of HeLa cells was 200 ng/ml (3 nM), based on Annexin V/PI and flow-cytometry analysis (Fig. 6B).

### Production of ETA bound-HuscFvs

**Figure 7:**
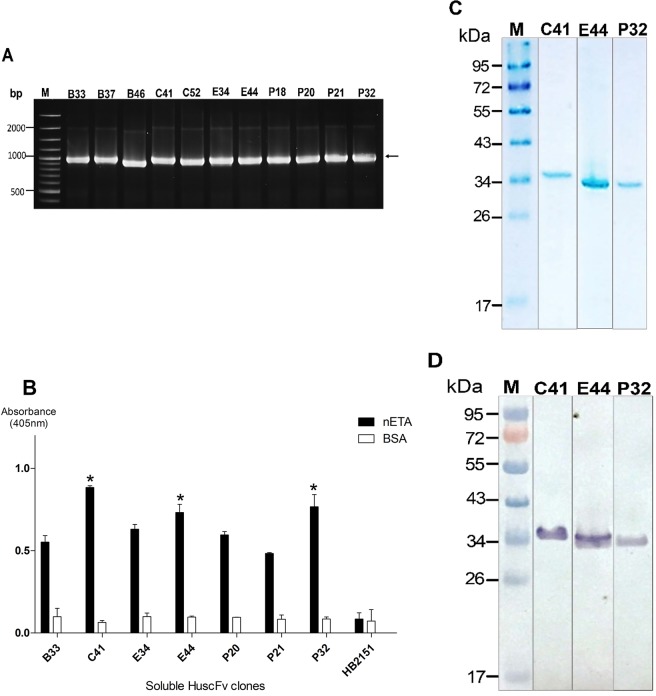
Production of ETA-bound HuscFvs. (**A**) Amplicons of HuscFv gene (~1,000 bp) from representative phage-transformed HB2151 *E. coli* clones. (**B**) Indirect ELISA results for determining the binding of HuscFvs in lysate of the representative phage-transformed-HB2151 *E. coli* clones to the native ETA (1 µg/well) and BSA (control antigen). (**C**) Stained SDS-PAGE-separated-purified and refolded rETA-bound HuscFvs (~34 kDa or slightly higher) from transformed NiCo21 (DE3) *E. coli* (1 μg/lane). (**D**) Western blot patterns of the representative ETA-bound HuscFvs from transformed NiCo21 (DE3) *E. coli* (1 μg/lane). Numbers at the left of A, DNA sizes in bp; numbers at the left of (**C,D**), protein molecular masses in kDa. Full-length blots/gels are presented in Supplementary Figs S2 and S3.

A total of 241 colonies of HB2151 *E. coli* transfected with ETA-bound phages were obtained from the HuscFv-phage display library by phage bio-panning using rETA-1A (binding domain; B), rETA-3 (catalytic domain; C), ETA-FL (E) and commercially synthesized biotinylated peptide of ETA catalytic site (ADAITGPEEEGGRLETILGW; P) as antigens; they were 61, 96, 51, and 33 clones from panning with B, C, E, and P, respectively. Of the 241 clones, 155 were positive for genes coding for HuscFvs (*huscfvs*), i.e., PCR amplicons at ~1,000 bp, as shown in Fig. 7A. Lysates of 37 of 155 clones produced soluble HuscFvs that bound to native ETA (Sigma, St. Louis, Mo., USA) using BSA as a control antigen; representative binders to native ETA are shown in Fig. 7B. After nucleotide sequencing, 16 clones (B33, B46, C41, C46, C48, C61, C83, E14, E34, E38, E40, E42, E44, P20, P21, and P32) showed complete sequences of single-chain antibodies, i.e., *huscfv* contained contiguous sequences coding for immunoglobulin (Ig)-VH, peptide linker (Gly_4_Ser1)_3_, and Ig-VL. These clones were categorized into 7 different types, based on the deduced amino-acid sequences and numbering of the Kabat and Chothia scheme^36^. They were type-1 (IGHV3 family and IGKV4 subgroup: P20 and E40), type-2 (IGHV1 family and IGKV1 subgroup: P21), type-3 (IGHV4 family and IGKV3 subgroup: E44), type-4 (IGHV3 family and IGKV3 subgroup: B33, B46, C46, C48, C61, and C83), type-5 (IGHV3 family and IGKV3 subgroup: C41 and E42), type-6 (IGHV1 family and IGKV3 subgroup: E34), and type-7 (IGHV3 family and IGKV2 subgroup: E14, E38, and P32). Subsequently, *huscfvs* of the *E. coli* clones C41, E44, and P32, whose HuscFvs showed high binding signals to native ETA by indirect ELISA (Fig. 7B; asterisks) were subcloned from pCANTAB5E phagemids into pLATE52 vectors and the recombinant plasmids were used to transform NiCo21 (DE3) *E. coli* for large-scale production of the respective HuscFvs. Inclusion bodies (IB), containing N-terminal 6× His-tagged-HuscFvs, were purified from homogenates of individual *E. coli* clones grown under IPTG induction, and refolded. The SDS-PAGE and Western blot patterns of purified HuscFvs of clones C41, E44, and P32 are shown in Fig. 7C,D, respectively. The quality of the HuscFv proteins recovered from the *E. coli* inclusion bodies was also estimated by circular dichroism. The protein secondary structures were revealed (Supplementary Fig. S5). By indirect ELISA, the refolded-recombinant HuscFvs retained the native ETA-binding activity of the original HuscFvs (Supplementary Fig. S6).

### HuscFvs protected mammalian cells from ETA-mediated cellular apoptosis

**Figure 8:**
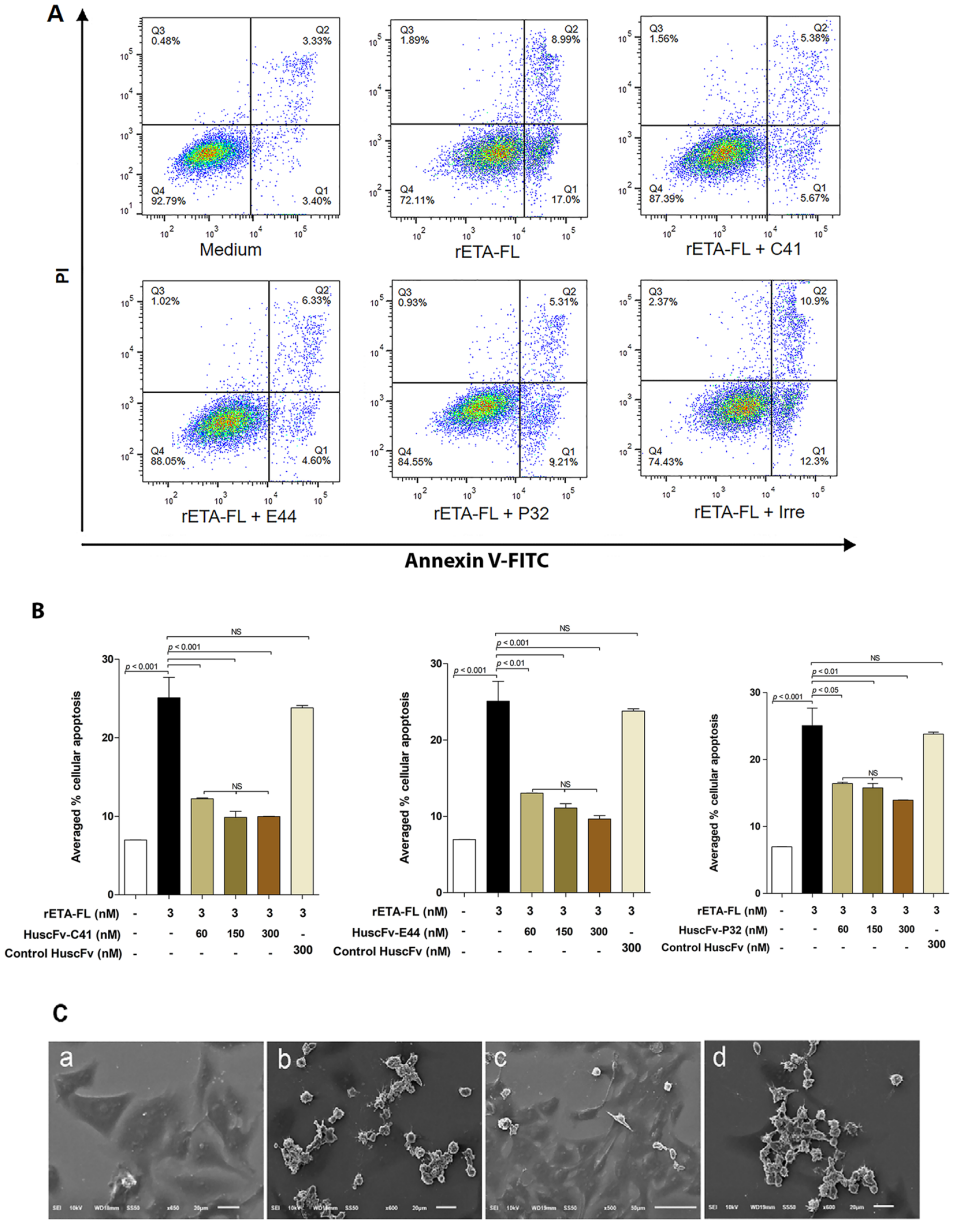
(**A**) Representative results of apoptotic analysis of Annexin V/PI stained HeLa cells exposed to ETA after treatment with ETA-bound HuscFvs (HuscFv 150 nM:rETA-FL 3 nM; molar ratio 50:1) using 300 nM control HuscFv (Irre) and medium alone as controls. (**B**) Bar graphs for statistical comparison of the averaged percentages of HeLa cell apoptosis induced by 3 nM ETA after treatments with 60, 150, and 300 nM HuscFvs (20:1, 50:1 and 100:1 molar ratio of HuscFv:ETA) using 300 nM control HuscFv and medium as controls. The data shown represent three independent experiments. A significant difference from the control is indicated by *p* < 0.05. (**C**) Scanning electron microscopic appearances of ETA-exposed HeLa cells after treatment with ETA-bound HuscFvs, control HuscFv (Irre) and medium alone. Normal HeLa cells (a); HeLa cells exposed to 3 nM ETA (b); cells exposed to 3 nM ETA treated with 150 nM HuscFv-C41 (as representative) (c); cells exposed to 3 nM ETA treated with 300 nM control HuscFv (d).

**Figure 9:**
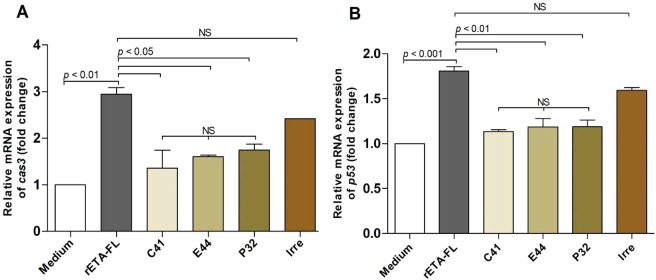
Expression of apoptosis-related genes (*cas3* and *p53*) as determined by real-time RT-PCR. A, the relative mRNA expression of *cas3*; B, the relative mRNA expression of *p*53 in 3 nM ETA-exposed HeLa cells treated with 150 nM ETA-bound-HuscFvs, 300 nM control HuscFv (Irre) and medium alone for 12 h. GAPDH mRNA was used as an internal control. Data from triplicated experiments.

Figure 8A shows the results for one of the three independent-experiments to demonstrate the protective effect of HuscFvs (150 nM) on 3 nM ETA-exposed cells. Annexin V/PI staining and flow cytometric analysis demonstrated that 3 nM ETA induced early apoptosis of mammalian (HeLa) cells by 16.53 ± 4.22% (quadrant 1; Annexin V+/PI--stained cells) and late apoptosis of the cells by 8.55 ± 0.46% (quadrant 2; Annexin V+/PI+-stained cells); hence, a total of 25.08 ± 4.51% of the apoptotic cells (quadrant 1 + quadrant 2; average of three-independent experiments). After treatment with 150 nM HuscFv-C41, HuscFV-E44, and HuscFv-P32 (representative HuscFv concentration), the average percentages of cellular apoptosis from the three-independent experiments was significantly reduced, to 9.85 ± 1.09, 11.09 ± 0.98, and 15.78 ± 1.13, respectively (Fig. 8A). The results of Annexin V/PI staining for apoptotic cells after treatment of the 3 nM ETA-exposed cells with different concentrations of individual HuscFvs (60, 150 and 300 nM) are shown in Table 1. Bar graphs for statistical comparisons of the averaged percentages of cellular apoptosis among the different treatment groups of the three independent experiments are shown in Fig. 8B. Control HuscFv (300 nM) showed averaged percent apoptotic cells at 23.8 ± 0.52%, which did not differ significantly from the ETA-exposed cells in medium alone (25.08 ± 4.51%). Normal HeLa cells had 6.97 ± 0.02% background apoptosis (average of three experiments, also). The ultrastructural studies by SEM confirmed that the HuscFvs (150 nM) could neutralize the ETA-induced HeLa cell apoptosis (Fig. 8C). The ETA-exposed HeLa cells treated with the HuscFvs showed significant decreases in the expression of apoptosis-related genes, including *cas3* and *p53*, compared with the control HuscFv-treated (Irre) cells and the cells in medium alone (Fig. 9A,B).

**Table 1:** Results of Annexin V-FITC/PI staining for apoptotic cells after treatment of the HeLa cells exposed to 3 nM rETA-FL with 60, 150, and 300 nM HuscFv-C41, HuscFv-E44 and HuscFv-P32, compared with ETA-exposed cells treated with 300 nM control HuscFv (Irre) (background control) and medium alone (negative control), and normal cells.

HeLa cells treated with	Percent cellular apoptosis	Percent cellular survival
Medium	6.97 ± 0.02	92.27 ± 0.25
rETA-FL	25.08 ± 4.51	73.03 ± 4.18
rETA-FL + 60 nM HuscFv- C41	12.21 ± 0.17	87.05 ± 0.49
rETA-FL + 150 nM HuscFv- C41	9.85 ± 1.09	89.07 ± 1.70
rETA-FL + 300 nM HuscFv- C41	9.96 ± 0.05	89.90 ± 0.42
rETA-FL + 60 nM HuscFv-E44	13.03 ± 0.03	86.25 ± 0.07
rETA-FL + 150 nM HuscFv-E44	11.09 ± 0.98	87.77 ± 0.97
rETA-FL + 300 nM HuscFv-E44	9.67 ± 0.63	89.75 ± 0.49
rETA-FL + 60 nM HuscFv-P32	16.42 ± 0.26	82.55 ± 0.07
rETA-FL + 150 nM HuscFv-P32	15.78 ± 1.13	83.27 ± 1.19
rETA-FL + 300 nM HuscFv-P32	13.94 ± 0.04	85.45 ± 0.07
rETA-FL + 300 nM control HuscFv (Irre)	23.80 ± 0.52	73.37 ± 0.93

Data represent the means ± standard deviations from three independent experiments.

### Computerized simulation for determining presumptive residues and domains of ETA that formed contact interface with the HuscFvs

To gain some insight into the mechanisms of the HuscFvs in rescuing cells from ETA-mediated cytotoxicity, a computerized simulation was conducted to predict the presumptive residues of the toxin interacted by the effective HuscFvs.

**Figure 10:**
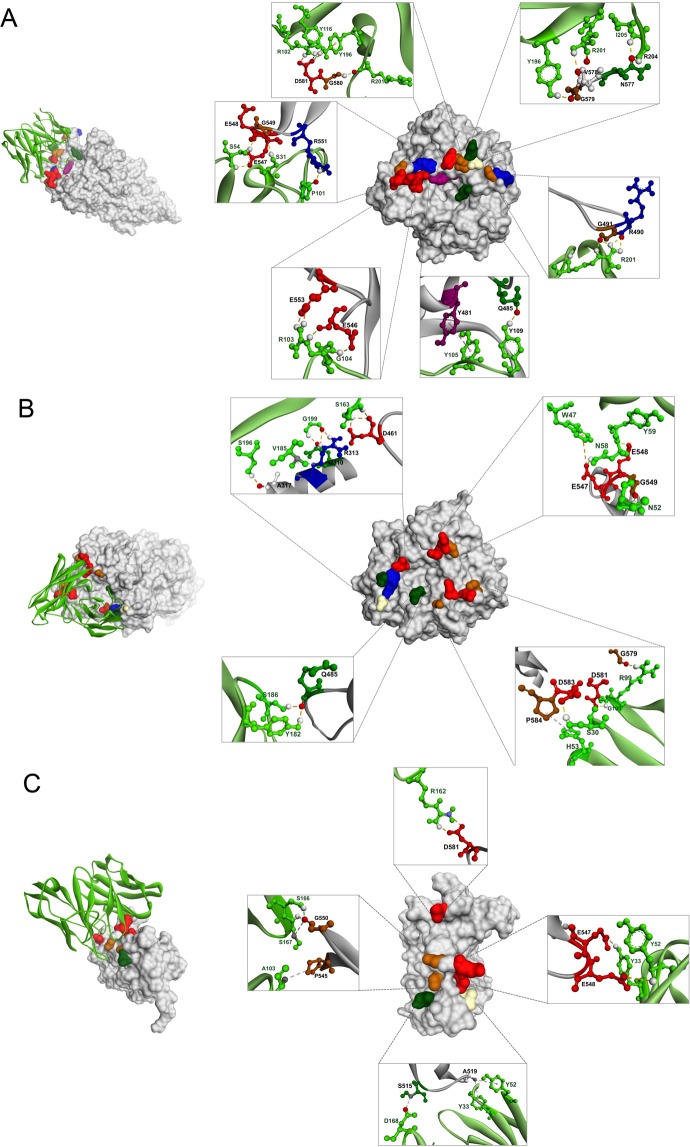
Computerized interaction of modeled-ETA and HuscFvs and residues that were predicted to form contact interfaces between them. Left side of Panels A–C, interactions of ETA (grey) with several residues near/or in the catalytic site of the respective HuscFvs (light green). Right side of Panels A–C, contact residues between ETA and HuscFv-C41, HuscFv-E44, and HuscFv-P32, respectively. The ETA amino acids are colored according to the CINEMA color scheme: polar negative D and E are red; polar positive H and R are blue; polar neutral S, N, and Q are dark green; non-polar aromatic Y is purple/magenta; non-polar aliphatic A and V are white, P and G are brown.

The three-dimensional (3D) structure of ETA (PDB 1IKQ) and modeled-HuscFvs were subjected to intermolecular docking. Figure 10 and Table 2 show the predicted ETA residues and domains that formed a contact interface with the HuscFvs of the *E. coli* clones C41, E44, and P32. By *in silico* docking, HuscFv-C41 uses VH-CDR1-3, VL-CDR2 as well as VL-FR2 and VL-FR3 to form contact with critical residues on the ETA-catalytic domain, including Y481, Q485, R490, G491, E546, E547, E548, G549, R551, E553, N577, V578, G579, G580, and D581, of which Y481, E546, E547, and E553, are NAD^+^ binding sites. E553 is the pivotal ETA residue for toxin enzymatic activity in transferring ADP-ribose to the eEF-2^37–39^. Y481 is the NAD^+^-binding site of the hydrophobic cavity of the toxin catalytic domain; ETA uses the phenol ring of Y481 to tether the nicotinamide ring of the NAD^+^ substrate, i.e., ADP-ribosyl group of NAD^+^, in order to transfer the substrate to eEF-2^24^. D461, Q485, E546, E547 and E553 formed the edge of the ETA-NAD^+^ binding cavity and participated in hydrogen bonding with two ribose moieties of the NAD^+^ molecule^40^. Moreover, a previous study indicated that the ETA epitopes recognized by murine monoclonal antibody clone B7 (MAbB7), which displayed strong neutralizing capacity on ETA cytotoxicity, were mapped to the C-terminal residues 575–595 of the toxin^41^. Although the results of ETA-HuscFv interaction by computerized simulation needs experimental validation, it is plausible that the effectiveness of HuscFv-C41 in neutralizing the apoptotic activity of ETA might be through interfering with the catalytic activity and inhibition of ADP-ribose transfer from NAD^+^ to eEF-2.

**Table 2:** Residues and domains of *P. aeruginosa* exotoxin A (ETA) that were predicted by computerized simulation to have interacted with the effective human single-chain antibodies, i.e., HuscFv-C41, HuscFV-E44 and HuscFv-P32.

Exotoxin A protein		HuscFv-C41		Interactive bond(s)
Residue	Domain	Residue	Domain(s)	
Y481	Catalytic (NAD^+^ binding site)	Y105	VH-CDR3	Hydrophobic (π-π stacking)
Q485	Catalytic	Y109	VH-CDR3	Hydrogen
R490	Catalytic	R201	VL-CDR2	Hydrogen
G491	Catalytic	R201	VL-CDR2	Hydrogen
E546	Catalytic (NAD^+^ binding site)	R103/G104	VH-CDR3	Hydrogen
E547	Catalytic (NAD^+^ binding site)	S54	VH-CDR2	Hydrogen
E548	Catalytic	S54	VH-CDR2	Hydrogen
G549	Catalytic	S31	VH-CDR1	Hydrogen
R551	Catalytic	P101	VH-CDR3	Hydrogen
E553	Catalytic (NAD^+^ binding site)	R103	VH-CDR3	Salt bridge
N577	Catalytic	G204/I205	VL-FR3	Hydrogen
V578	Catalytic	R201	VL-CDR2	Hydrogen
G579	Catalytic	Y196	VL-FR2	Hydrogen
G580	Catalytic	R201	VL-CDR2	Hydrogen
D581	Catalytic	R102/Y116/Y196	VH-CDR3/VH-CDR3/VL-FR2	Salt bridge/Hydrogen/Anionic π
**Exotoxin A protein**		**HuscFv-E44**		**Interactive bond(s)**
**Residue**	**Domain**	**Residue**	**Domain(s)**	
Q310	Translocation (ETA-2)	G199	VL-FR3	Hydrogen
R313	Translocation	V185/G199	VL-CDR2/VL-FR3	Hydrophobic (alkyl)/Hydrogen
A317	Translocation	S196	VL-FR3	Hydrogen
D461	Catalytic (NAD^+^ binding site)	S163	VL-CDR1	Hydrogen
Q485	Catalytic	Y182/S186	VL-FR2/VL-CDR2	Hydrogen
E486	Catalytic	S186	VL-CDR2	Hydrogen
E547	Catalytic (NAD^+^ binding site)	W47/N58	VH-FR2/VH-CDR2	Anionic π/Hydrogen
E548	Catalytic	Y59	VH-CDR2	Hydrogen
G549	Catalytic	N52/R99	VH-CDR2/VH-CDR3	Hydrogen
D581	Catalytic	G100	VH-CDR3	Hydrogen
D583	Catalytic	S30	VH-FR1	Hydrogen
P584	Catalytic	H53	VH-CDR2	Hydrophobic (π-alkyl)
**Exotoxin A protein**		**HuscFv-P32**		**Interactive bond(s)**
**Residue**	**Domain**	**Residue**	**Domain(s)**	
S515	Catalytic	D168	VL-CDR1	Hydrogen
A519	Catalytic	Y33/Y52	VH-CDR1/VH-CDR2	Hydrophobic (π-alkyl)
P545	Catalytic	A103	VH-CDR3	Hydrophobic (alkyl)
E547	Catalytic (NAD^+^ binding site)	Y33	VH-CDR1	Hydrogen
E548	Catalytic	Y52	VH-CDR2	Hydrophobic (π-alkyl)
G550	Catalytic	S166/S167	VL-CDR1	Hydrogen
D581	Catalytic	R162	VL-CDR1	Salt bridge

HuscFv-E44 was predicted to use VL-CDR2 and VL-FR3 to interact with Q310, R313, and A317 of ETA-2 (translocation domain); this binding should not be involved in the HuscFv-mediated-neutralization of ETA cytotoxicity. The HuscFv-E44 also used VH-CDR2, VH-CDR3, VH-FR1, VL-CDR1, VL-CDR2, and VL-FR2, to bind to D461 and E547, which are NAD^+^ binding sites, and Q485, E486, E548, G549, D581, D583, and P584 of ETA-3 of the catalytic domain. HuscFv-P32 interacted with residues of the ETA-catalytic domain, including S515, A519, P545, E547 (NAD^+^ binding site), E548, G550, and D581, by using VH-CDR1-3 and VL-CDR1. Based on the computerized results of protein-antibody docking, the ETA bound-HuscFvs of the three *E. coli* clones presumptively formed contact interfaces with the critical residues for ETA-mediated ADP-ribosylation. Nevertheless, the possibility also exists that the effective HuscFvs might inhibit/interfere with some other steps of ETA-mediated cellular intoxication, either by steric hindrance of-, or direct binding to-, the toxin critical residues and in effect causing interference with the respective toxin bioactivity(-ies) such as receptor binding, clathrin-dependent cellular internalization, endosomal exit, retrograde transportation of the toxin through the Golgi network, or EF2-ADP-ribosylation. Experiments are needed to pinpoint the mechanism(s) of the effective HuscFvs. For this study, human single-chain antibodies that effectively neutralized ETA activity were successfully generated. Besides elucidation of their molecular mechanisms of ETA inhibition, these antibodies should be tested further, step-by-step, towards clinical use (e.g., GMP production, pre-clinical trial, as well as different phases of clinical trials), as a safe immunotherapeutic against ETA of difficult-to-treat *P. aeruginosa* infections.

## Materials and Methods

### Cell culture

In this study, HeLa cells (human cervical epithelial cancer cells) were grown in Dulbecco’s Modified Eagle’s Medium (DMEM) supplemented with 10% fetal bovine serum (FBS) and 1% penicillin/streptomycin at 37 °C in a humidified 5% CO_2_ atmosphere.

### Production of recombinant ETA domain-1A (rETA-1A), domain-3 (rETA-3), and full-length-ETA (rETA-FL)

In this study, the synthesized DNA of *P. aeruginosa* ETA (GenScript, New Jersey, USA) was used as the template to amplify the ETA genes–*ETA-1A*, *ETA-3*, and *ETA-FL–*by PCR using Phusion High-Fidelity DNA polymerase (Thermo Fisher Scientific). Specific primers for the respective gene amplification were designed according to the deduced amino acids of *P. aeruginosa* full-length ETA gene sequence (Gene Bank accession no: NC_002516.2) (Supplementary Table S2). The PCR amplicons were inserted appropriately into the pLATE52 expression vector, and the recombinant vectors were introduced into the expression host, i.e., NiCo21 (DE3) *E. coli* (New England Biolabs, UK). The appropriately transformed bacterial colonies were grown in LB broth containing 100 μg/ml ampicillin until an OD_600nm_ was 0.5; then expressions of the recombinant proteins were induced by adding 1 mM isopropyl-β-D-1-thiogalactopyranoside (IPTG) to individual cultures at 37 °C for 4 h. Thereafter, the bacterial cells were harvested by centrifugation and lysed using BugBuster^®^ Protein Extraction Reagent (Novagen, Merck KGaA, Darmstadt, Germany) supplemented with 10 microliters of Lysonase™ Bioprocessing Reagent (Novagen) per gram of bacterial cell pellet. The bacterial inclusion bodies (IBs) containing the recombinant proteins in the pellets were collected after centrifugation of the *E. coli* homogenates. The IBs were washed sequentially with Wash-100 buffer [50 mM sodium phosphate buffer, pH 8.0; 500 mM NaCl; 5 mM EDTA; 8% w/v glycerol and 1% v/v Triton X-100], Wash-114 buffer [50 mM Tris buffer, pH 8.0; 300 mM NaCl and 1% v/v Triton X-114], Wash-Solvent buffer [50 mM Tris buffer, pH 8.0 and 60% v/v isopropanol], and finally with Milli-Q^®^ water (Merck Millipore). After centrifugation, small volumes of ultrapure-water were added to the collected pellets and stored at 4 °C. The quality of the preparations was analyzed by a 12% SDS-PAGE and Western blotting and the quantities determined by Pierce™ BCA Protein Assay Kit (Thermo Fisher Scientific, Rockford, lL, USA). LC-MS/MS was used to verify the recombinant proteins. Afterwards, the purified IBs containing rETA-1A, rETA-3, and rETA-FL, were solubilized separately at 0.5 mg/ml in 5 ml of 50 mM CAPS, pH 11.0; 0.3% w/v N-lauryl sarcosine and 1 mM DTT, and kept at 4 °C until completely dissolved. The proteins were refolded by dialysis at 4 °C with slow stirring against 750 ml of 20 mM Tris-HCl, pH 8.5 supplemented with 0.1 mM DTT for 3 h, then the dialysis buffer was changed to 20 mM Tris-HCl, pH 8.5 containing 0.1 mM DTT, at 4 °C overnight. The refolded ETA-1A and ETA-3 were finally dialyzed against 20 mM Tris-HCl, pH 8.5, while the ETA-FL preparation was dialyzed against 20 mM imidazole, pH 8.5. All preparations were then filtered through 0.2 µm low-protein-binding Acrodisc^®^ syringe filter (Pall, Port Washington, NY, USA). The protein contents were determined using a BCA assay before keeping in 8% (w/v) glycerol at −80 °C until use.

Folding of the refolded-rETA-1A, rETA-3 and rETA-FL was determined by using Far-UV Circular Dichroism (CD) measurements. Briefly, 400 μl of the proteins (0.25 mg/ml) were subjected for CD measurements on a JASCO J-815 spectropolarimeter equipped with Peltier temperature controller system (Jasco Co., Ltd., Tokyo, Japan). The refolded protein spectra were recorded in a 0.1 cm path-length quartz cuvette in 20 mM Tris-HCl, pH 8.5. The proteins were scanned from 190 and 260 nm at a speed of 50 nm/min at 25 °C. An average of three scans was used to generate the CD spectra for each protein.

### Biological activities of rETA-FL

Morphological characteristics of HeLa cells treated with 3 nM of rETA-FL were evaluated by electron microscopy (EM) and dual AO/EB staining. For AO/EB staining, cells were trypsinized, washed three times with PBS, pH 7.4 and the pellets were resuspended in 50 μl of culture medium. The cells were stained in an ice-cold container with 100 μg/ml of AO and 100 μg/ml of EB [cell:dye ratio 2:1 (v/v)]. The stained cell suspensions were dropped onto glass slides, covered with coverslips, and observed under a fluorescence microscope.

SEM and TEM were used to study the ultrastructure of the cells after exposure to ETA, and the ETA-exposed cells treated with HuscFvs. For SEM, the HeLa cells that had been grown on coverslips for 24 h were fixed with 2.5% glutaraldehyde at room temperature for 1 h, washed with sucrose phosphate buffer (SPB) three times, followed by fixing with 1% osmium tetroxide in 0.1 M SPB for 1 h and washed again. They were then dehydrated in ethanol gradients, and allowed to air-dry overnight. The coverslips were mounted on an aluminum stub and coated with a gold film (20 nm-thickness) using a sputter coater (Emitech K550, Ashford, UK). They were examined under a scanning electron microscope (JEOL JSM-6610LV, Japan) with 15 kV acceleration voltages. For TEM, the ethanol-dehydrated cells were infiltrated with LR white embedded medium (EMS, USA.) in 70% ethanol, then embedded in a capsule beam and kept in a 65 °C incubator for 48 h. Ultra-thin sections (90–100 nm thickness) of the preparations were placed on a 200 square-mesh copper grid and stained with uranyl acetate and lead citrate. Fine cell morphology was examined under a transmission electron microscope (Hitachi HT7700, Japan).

### Flow cytometry analysis

Apoptotic cells were revealed by double-staining with Annexin V/PI using an FITC-Annexin V Apoptosis Detection Kit (BD Biosciences) according the manufacturer’s instructions. HeLa cells (1 × 10^5^ cells in 500 µl completed culture medium) were incubated with different concentrations of rETA-FL (200–2,000 ng/ml) for 24  h. Cells in medium alone were included in the experiments. After incubation, the cells were trypsinized, harvested, and washed with ice-cold PBS, pH 7.4. One hundred microliters of the cell suspension (∼1 × 10^5^ cells) were transferred to a 5-ml culture tube. The cells were stained by adding 5 μl each of FITC-Annexin V and PI working solutions and incubated at 25 °C for 15 min in the dark. The preparation was diluted with 500 μl of 1× binding buffer and the stained cells were immediately analyzed by flow cytometry. To determine the minimum concentration of rETA-FL that induced HeLa cell apoptosis, the HeLa cells were treated with different concentrations of rETA-FL and percent cellular apoptosis was determined for each rETA-FL concentration.

### Production of ETA-bound HuscFvs

The refolded rETA-1A, rETA-3, rETA-FL, and commercially synthesized (GenScript, New Jersey, USA) biotinylated peptide containing ETA catalytic residues (biotin-6-aminohexanoic acid-ADAITGPEEEGGRLETILGW) were used as panning antigens. The refolded rETA-1A, rETA-3, and rETA-FL (0.5 µg in 100 μl of coating buffer) were used to coat separate wells of EIA/RIA 8-well strips and kept at 4 °C overnight. For biotinylated-ETA peptide, 2.5 µM of the peptide in 100 μl PBS were added to well of a streptavidin plate (Pierce™ Streptavidin Coated Plates, Clear Well Strips, Rockford, IL, USA) and kept at room temperature for 1 h. After blocking with Pierce™ Protein-Free (PBS) Blocking Buffer (Thermo Fisher Scientific, Rockford, IL, USA) and washed with PBS containing 0.01% Tween-20 (PBST), the previously constructed HuscFv-phage display library^42^ was added to individual antigen coated wells. Binding of HuscFv-display phages to the immobilized antigens were allowed at room temperature for 1 h on a rocking platform. Antigen-unbound phages were removed by washing thoroughly with PBST. Aliquots of log phase-grown HB2151 *E. coli* culture were added to each phage-containing well. In the case of the biotinylated-ETA peptide bound phages, 50 µM of peptide were added for competitive elution of the phages before adding to the bacteria. The phage-transformed *E. coli* were plated onto the 2× YT agar containing 2% glucose and 100 μg/ml ampicillin. After overnight incubation, the bacterial colonies grown on the selective agar were screened for clones carrying pCANTAB5E-*huscfv* phagemids by colony PCR using R1 (forward primer): 5′-CCA TGA TTA CGC CAA GCT TTG GAG CC-3′ and R2 (reverse primer): 5′-GCT AGA TTT CAA AAC AGC AGA AAG G-3′. The *E. coli* clones with *huscfv* amplicons (∼1,000 bp) were grown in 2 ml of auto-induction medium containing 100 µg/ml ampicillin at 30 °C with shaking at 250 rpm, overnight. The presence of HuscFvs in the *E. coli* homogenates was checked by Western blotting using anti-E tag antibody as the E-tagged-HuscFv tracer. *E. coli* homogenates containing soluble HuscFvs were tested for binding to native ETA (Sigma, St. Louis, Mo., USA) (test antigen) by indirect ELISA using BSA as the control antigen. *E. coli* clones that the HuscFvs in their homogenates gave an ELISA signal at OD_405nm_ above mean + 3 SD of the background binding control (lysate of original *E. coli* HB2151) and more than two times higher than to the control antigen, were selected. Nucleotides of the *huscfvs* coding for the ETA-bound-HuscFvs were sequenced, deduced, and the complementarity-determining regions (CDRs) and their respective canonical immunoglobulin framework regions (FRs) were determined using an online server, the VBASE2- the integrative germ-line V gene database (http://www.vbase2.org/).

To produce ETA-bound HuscFvs on a large scale, the *huscfvs* of the selected HB2151 *E. coli* were subcloned from the pCANTABE5E phagemids to the pLATE52^TM^ expression vector using a ligation independent cloning (LIC) system (Thermo Fisher Scientific) and the recombinant plasmids were introduced into JM109 *E. coli*. The recombinant pLATE52-*huscfv* plasmids were individually used to further transform into NiCo21 (DE3) *E. coli*. The selected transformed bacterial colonies were grown under 1 mM IPTG induction in LB medium containing 100 µg/ml ampicillin and each bacterial pellet was suspended in BugBuster^®^Protein Extraction buffer (5 ml/g bacterial wet weight) (Novagen) supplemented with 20 μl of Lysonase™ Bioprocessing Reagent (Novagen) and kept at 25 °C with agitation. To each preparation, Lysonase^TM^ Bioprocessing reagent (10 µl/g of bacteria) was added, with further agitation for 20 min. Inclusion bodies (IBs) were harvested by centrifugation at 8,000 × *g* at 4 °C for 30 min, washed twice with Wash-100 solution, twice with Wash-114 buffer and once with Wash-Solvent. The preparations were shaken vigorously during the washings, followed by centrifugation, as above. The 20 ml of Milli-Q^®^ water (Merck Millipore) were added to the preparations, which were then placed on a shaker at 25 °C for 20 min, and centrifuged at 8,000 × *g* for 20 min. The IBs were solubilized in buffer [50 mM CAPS, pH 11.0; 0.3% (w/v) N-lauryl sarcosine; 1 mM DTT] and kept at 4 °C for 16 h or until completely dissolved. Each preparation was dialyzed in Slide-A-Lyzer^®^ 2 K Dialysis Cassettes G2 (Thermo Fisher Scientific, Rockford, IL, USA) at 4 °C with slow stirring against refolding buffer [20 mM imidazole, pH 8.5, supplemented with 0.1 mM DTT], filtered through 0.02 µm low protein binding Acrodisc^®^ syringe filter (Pall, Port Washington, NY, USA), and kept in water bath at 30 °C for 3 h before 60 mM trehalose were added. Protein quantity and quality were determined using a Pierce BCA™ Protein Assay (Thermo Fisher Scientific) and SDS-PAGE and protein staining, respectively. All preparations were stored at −80 °C until use. The refolded-HuscFvs were retested for binding to native ETA by indirect ELISA.

### Computerized simulation for determining presumptive residues and domains of ETA bound by the HuscFvs

Homology modeling and intermolecular docking were used to predict the presumptive residues of the ETA bound by the HuscFvs. The three-dimensional (3D) structure of wild-type *P. aeruginosa* ETA was retrieved from RCSB PDB 1IKQ. The 3D structures of the ETA-bound-HuscFvs were modeled by the I-TASSER server^43,44^. The physical quality of each I-TASSER predicted 3D model was further refined by high-resolution protein structure refinement, i.e., ModRefiner^45^. Subsequently, their low free-energy conformations, which were closer to their native structures, were improved by removing steric clashes and improving the torsion angles and hydrogen-binding networks by molecular dynamics (MD) -based algorithm, full-atomic simulations using Fragment Guide Molecular Dynamics Simulation (FG-MD)^46^. The ETA-HuscFv complexes were built on the automated ClusPro 2.0 antibody-protein docking server^47^. All models were analyzed and visualized using the program Discovery studio 3.5 and PyMol software (PyMol Molecular Graphics System, Version 2 edu, Schrodinger, LLC).

### Determination of HuscFv capability in rescuing cells from ETA-mediated apoptosis

For staining and flow cytometric analysis, ETA-exposed HeLa cells were stained by annexin V/PI and analyzed by flow cytometry, as described above. Ultrastructural studies of the cells of all treatments were investigated by SEM.

### Detection of expression of apoptosis-related genes (*cas3* and *p53*) by quantitative reverse transcription-polymerase chain reaction (qRT-PCR)

Total RNA was extracted from the ETA-exposed HeLa cells that had been treated with HuscFvs and controls for 12 h, using a GeneJET RNA Purification Kit (Thermo Fisher Scientific). The quality of the RNAs was determined at OD_260nm_ and OD_280nm_ by NanoDrop™ 2000/2000c Spectrophotometer. After DNase treatment using RNase-free-DNase I (Thermo Fisher Scientific), the RNAs were used to generate cDNA by RevertAid First Strand cDNA Synthesis Kit (Thermo Fisher Scientific). Real-time RT-PCR was performed on KAPA SYBR^®^ FAST qPCR (Kapa Biosystems, Cape Town, South Africa). Each reaction mixture (20 µl) contained 10 µl of 2 × KAPA SYBR^®^ FAST qPCR Master Mix Universal, 200 nM final concentration each of forward and reverse primers, and 20 ng of cDNA template in nuclease-free PCR-grade water. The reaction was performed in a CFX96 Touch™ Real-Time PCR Detection System (Bio-Rad Laboratories). The following thermal cycles was used: initial denaturation at 95 °C for 3 min and 40 cycles at 95 °C for 30 s, 53 °C for 30 s, and 72 °C for 30 s. A dissociation curve was generated from a thermal profile consisting of 95 °C for 1 min, 55 °C for 30 s, and 95 °C (0.5 °C/s). Each sample was amplified in triplicate. Gene expressions relative to normal cells were analyzed using the ΔC_T_ method. The amounts of *casp3* and *p53* were normalized to the internal control, i.e., GAPDH. The primers used in these experiments are shown in Supplementary Table S2.

### Statistical analysis

GraphPad Prism version 5 (La Jolla, CA, USA) was used to compare the results of all tests. Statistically significant differences were determined by one-way ANOVA and Bonferroni test. *P*-value < 0.05 was considered statistically significant.

## Supplementary information

Revised supplementary information

## Data Availability

The datasets generated during the present study are accessible from the corresponding author on reasonable request.
