# Supplementary material for: Human single-chain antibodies that neutralize Pseudomonas aeruginosa-exotoxin A-mediated cellular apoptosis
Source: Sci Rep. 2019 Oct 17;9:14928. doi: 10.1038/s41598-019-51089-w (PMC6797803; doi:10.1038/s41598-019-51089-w)
Supplement: Supplementary file 1 — Revised supplementary information [file 41598_2019_51089_MOESM1_ESM.pdf]

Human single-chain antibodies that neutralize *Pseudomonas aeruginosa*-exotoxin A-mediated cellular apoptosis

Sirijan Santajit[1], Watee Seesuay[2], Kodchakorn Mahasongkram[2], Nitat Sookrung[2,3], Sumate Ampawong[4], Onrapak Reamtong[5], Pornphan Diraphat[6], Wanpen Chaicumpa[2], Nitaya Indrawattana[1]

[1]Department of Microbiology and Immunology, [4]Department of Tropical Pathology, and [5]Department of Tropical Molecular Biology and Genetics, Faculty of Tropical Medicine, Mahidol University, Bangkok, Thailand. [2]Center of Research Excellence on Therapeutic Proteins and Antibody Engineering, Department of Parasitology, Faculty of Medicine Siriraj Hospital, Mahidol University, Bangkok, Thailand. [3]Biomedical Research Incubator Unit, Department of Research, Faculty of Medicine Siriraj Hospital, Mahidol University, Bangkok, Thailand. [6]Department of Microbiology, Faculty of Public Health, Mahidol University, Bangkok, Thailand. Correspondence and requests for materials should be addressed to N.I. (email: nitaya.ind@mahidol.ac.th).

Protein	Orthologous protein	Accession no.	Protein score	Peptide score	Matched peptide sequence
rETA-1A	Chain A, *Pseudomonas aeruginosa* Exotoxin A, Wild Type	Gi 17943391	744	40.79	VLAGNPAK
				24.66	DATFFVR
				38.44	WSEWASGK
				30.42	RWSEWASGK
				47.66	LARDATFFVR
				88.81	ACVLDLKDGVR
				84.14	LEGGVEPNKPVR
				60.38	HDLDIKPTVISHR
				77.18	CNLDDTWEGKIYR
				105.82	VLCLLDPLDGVYNYLAQQR
				51.09	GSWSLNWLVPIGHEKPSNIK
				28.86	VLAGNPAKHDLDIKPTVISHR
				65.75	AHESNEMQPTLAISHAGVSVVMAQAQPR

Protein	Orthologous protein	Accession no.	Protein score	Peptide score	Matched peptide sequence
rETA-3	Exotoxin A [*Pseudomonas aeruginosa*]	Gi 156255901	652	23.48	IRNGALLR
				27.96	SSLPGFYR
				35.21	LIGHPLPLR
				47.27	GTQNWTVER
				46.7	SQDLDAIWR
				42.82	ARSQDLDAIWR
				56.98	TVVIPSAIPTDPR
				50.58	LLQAHRQLEER
				30.87	LETILGWPLAER
				56.1	TGLTLAAPEAAGEVER
				97.72	GFYIAGDPALAYGYAQDQEPDAR
				37	SSLPGFYRTGLTLAAPEAAGEVER
				72.84	LDAITGPEEEGGRLETILGWPLAER

Protein	Orthologous protein	Accession no.	Protein score	Peptide score	Matched peptide sequence
rETA-FL	Exotoxin A [*Pseudomonas aeruginosa*]	Gi 489172780	1019	40.59	VLAGNPAK
				20.32	ACVLDLK
				29.48	DATFFVR
				41.03	SSLPGFYR
				33.59	WSEWASGK
				67.59	LVALYLAAR
				35.24	LIGHPLPLR
				42.25	GTQNWTVER
				65.01	SQDLDAIWR
				39.76	RWSEWASGK
				93.17	LALTLAAAESER
				55.4	ACVLDLKDGVR
				82.08	LEGGVEPNKPVR
				49.23	ARSQDLDAIWR
				65.98	LSWNQVDQVIR
				58.08	TVVIPSAIPTDPR

				53.3	HDLDIKPTVISHR
				101.72	NALASPGSGGDLGEAIR
				85.86	TGLTLAAPEAAGEVER
				47.16	LALTLAAAESERFVR
				51.12	CNLDDTWEGKIYR
				77.83	GWEQLEQCGYPVQR
				26.83	LAIDNALSITSDGLTIR
				99.21	NYPTGAEFLGDGGDISFSTR
				19.44	VLAGNPAKHDLDIKPTVISHR
				50.01	NALASPGSGGDLGEAIREQPEQAR
				102.28	GFYIAGDPALAYGYAQDQEPDAR
				69.51	LDAITGPEEEGGRLETILGWPLAER

Supplementary Table S1. LC-MS/MS Mascot results of peptides generated from in-gel tryptic digestion of rETA-1A, rETA-3 and rETA-FL searching against the NCBInr database.

Primer	Primer sequence (5´- 3´)
ETA-1A forward	GGT TGG GAA TTG CAA GCG GAA GAG GCG TTT GAT TTG TG
ETA-1A reverse	GGA GAT GGG AAG TCA TTA TTC TGG AAA GTG CAG GCG GTG
ETA-3 forward	GGT TGG GAA TTG CAA GGT GAT ATT AGC TTT AGC ACC CGC GG
ETA-3 reverse	GGA GAT GGG AAG TCA TTA TTT CAG ATC TTC ACG TGG CGG C
ETA-FL forward	GGT TGG GAA TTG CAA GCG GAA GAG GCG TTT GAT TTG TG
ETA-FL reverse	GGA GAT GGG AAG TCA TTA TTT CAG ATC TTC ACG TGG CGG C
cas3 forward	TGG TTC ATC CAG TCG CTT TG
cas3 reverse	ATT CTG TTG CCA CCT TTC G
p53 forward	ACT AAG CGA GCA CTG CCC AA
p53 reverse	ATG GCG GGA GGT AGA CTG AC
GAPDH forward	CTG GGC TAC ACT GAG CAC C
GAPDH reverse	AAG TGG TCG TTG AGG GCA ATG

Supplementary Table S2. Primers used in this study.

Protein name	C-score	TM-score	RMSA (Å)	No. of decoys	Cluster density
HuscFv-C41	0.70	0.81±0.09	4.4±2.9	8787	0.5128
HuscFv-E44	0.89	0.83±0.08	3.9±2.7	9517	0.6571
HuscFv-P32	0.68	0.81±0.09	4.4±2.9	9727	0.5100

Supplementary Table S3. Estimated accuracy of the modeled HuscFvC41, HuscFvE44, and HuscFvP32 from I-TASSER.

ETA-HuscFv	Complex name	ΔG (kcal/mol)	Kd (M)
ETA-C41	model.000.00	-8.3	7.50E-07
	model.000.01	-9.6	8.60E-08
	model.000.02	-8.3	7.60E-07
	model.000.03	-9.6	8.80E-08
	model.000.04	-7.8	1.80E-06
	model.000.05	-9.7	7.20E-08
	model.000.06	-10.1	3.70E-08
	model.000.07	-9.3	1.50E-07
	model.000.08	-10.3	2.60E-08
	model.000.09	-10.2	3.20E-08
	model.000.10	-10.7	1.40E-08
	model.000.11	-11.7	2.60E-09
	model.000.12	-10.5	1.90E-08
	model.000.13	-8.3	8.20E-07
	model.000.14	-11.2	6.60E-09
	model.000.15	-7.8	1.70E-06
	model.000.16	-10	4.80E-08
	model.000.17	-8.3	8.10E-07
	model.000.18	-8.8	3.60E-07
	model.000.19	-10.6	1.60E-08
	model.000.20	-11.6	3.10E-09
	model.000.21	-8.7	3.90E-07
	model.000.22	-11	8.90E-09
	model.000.23	-9.3	1.60E-07
	model.000.24	-13.6	9.90E-11
	model.000.25	-9.3	1.60E-07
	model.000.26	-9.6	8.40E-08
ETA-E44	model.000.00	-9.9	5.10E-08
	model.000.01	-11.7	2.50E-09
	model.000.02	-7.7	2.20E-06
	model.000.03	-10.8	1.10E-08
	model.000.04	-12.6	5.90E-10
	model.000.05	-9.4	1.30E-07
	model.000.06	-12.2	1.10E-09
	model.000.07	-10.6	1.70E-08
	model.000.08	-11.4	4.50E-09
	model.000.09	-10.9	1.10E-08
	model.000.10	-9.1	2.20E-07
	model.000.11	-11.3	5.50E-09
	model.000.12	-16.1	1.60E-12
	model.000.13	-7.8	1.90E-06
	model.000.14	-9	2.50E-07
	model.000.15	-9.7	7.90E-08
	model.000.16	-11.1	7.20E-09
	model.000.17	-12.6	6.10E-10
	model.000.18	-10.4	2.30E-08
	model.000.19	-9	2.40E-07

	model.000.20	-11	8.20E-09
	model.000.21	-9.6	9.70E-08
	model.000.22	-15.4	5.00E-12
	model.000.23	-10.1	3.80E-08
	model.000.24	-8.1	1.20E-06
	model.000.25	-14	5.10E-11
	model.000.26	-8.3	7.60E-07
	model.000.27	-8.4	7.30E-07
	model.000.28	-10.1	4.10E-08
	model.000.29	-10.7	1.40E-08
ETA-P32	model.000.00	-5.5	9.80E-05
	model.000.01	-7.2	5.30E-06
	model.000.02	-6.7	1.10E-05
	model.000.03	-10.4	2.20E-08
	model.000.04	-6.3	2.50E-05
	model.000.05	-7.8	2.00E-06
	model.000.06	-9.2	1.90E-07
	model.000.07	-4.8	3.10E-04
	model.000.08	-5.1	2.00E-04
	model.000.09	-6.1	3.10E-05
	model.000.10	-8.8	3.60E-07
	model.000.11	-6.3	2.20E-05
	model.000.12	-8	1.40E-06
	model.000.13	-9.7	7.70E-08
	model.000.14	-5.8	5.40E-05
	model.000.15	-7.4	3.90E-06
	model.000.16	-9	2.60E-07

Supplementary Table S4. The binding affinity (ΔG) and dissociation constant (Kd) predicted values of ETA bound to individual HuscFv.

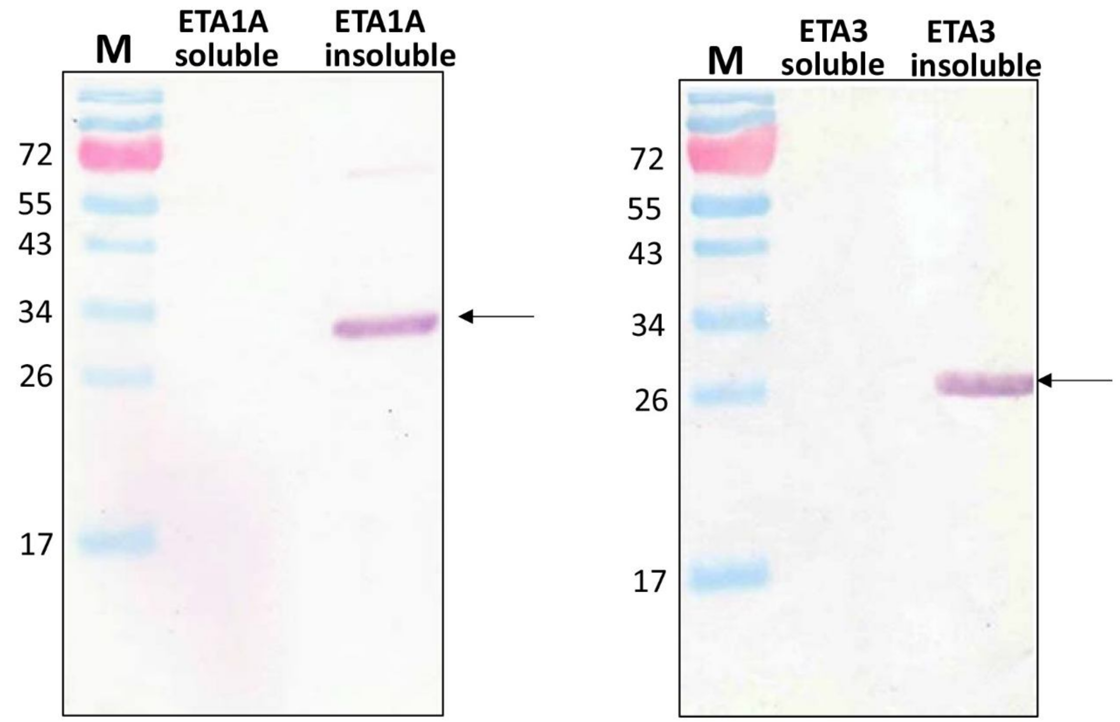

Supplementary Figure S1. Production of recombinant ETA-1A, ETA-3 and ETA-FL. Panel C of Figure 1, Western blot patterns of rETA-1A (28 kDa) and rETA-3 (26 kDa).

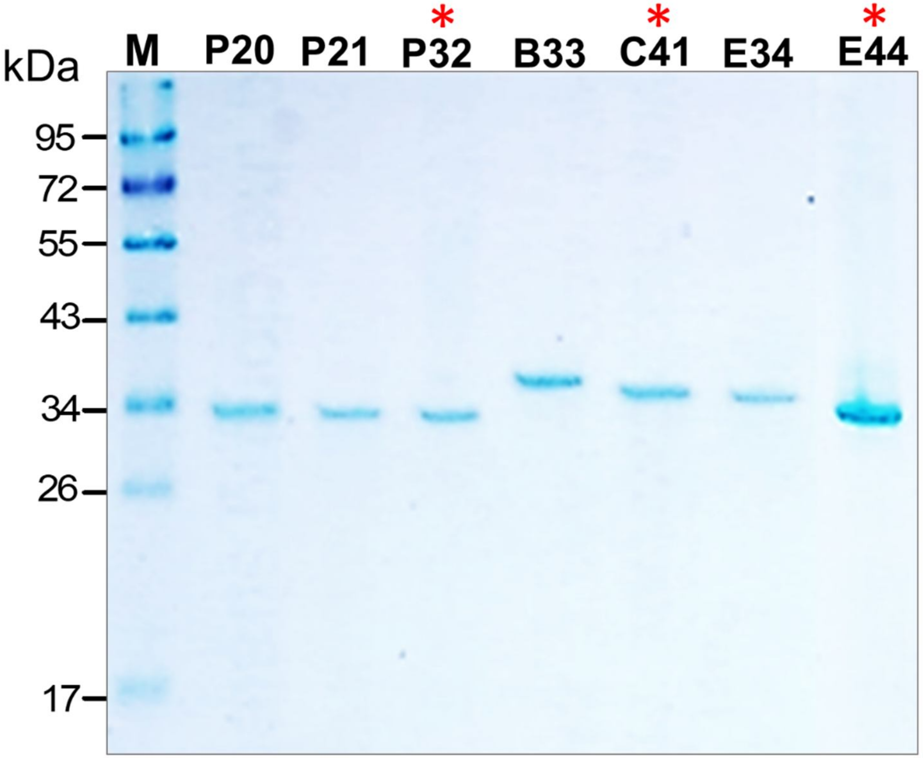

Supplementary Figure S2. Panel C of Figure 7, Stained SDS-PAGE-separated-purified and refolded rETA-bound HuscFvs (~34 kDa or slightly higher) from transformed NiCo21 (DE3) *E. coli* (1 µg per lane).

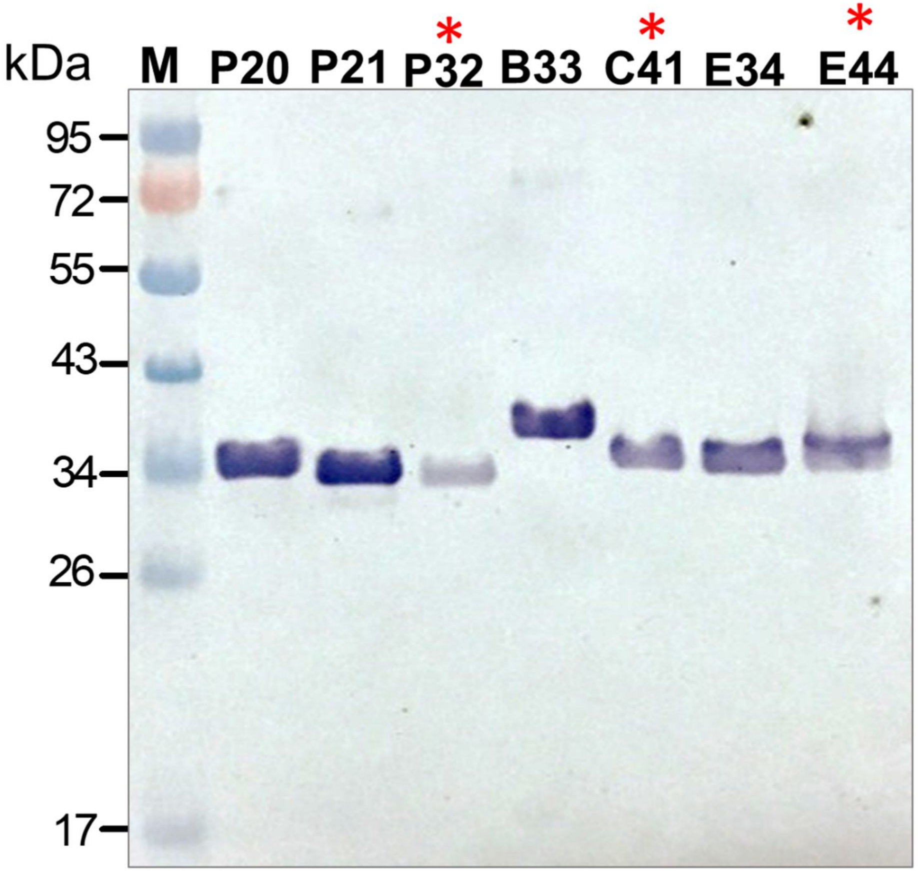

Supplementary Figure S3. Panel D of Figure 7, Western blot patterns of the representative ETA-bound HuscFvs from transformed NiCo21 (DE3) *E. coli* (1 µg per lane). Numbers at the left, protein molecular masses in kDa.

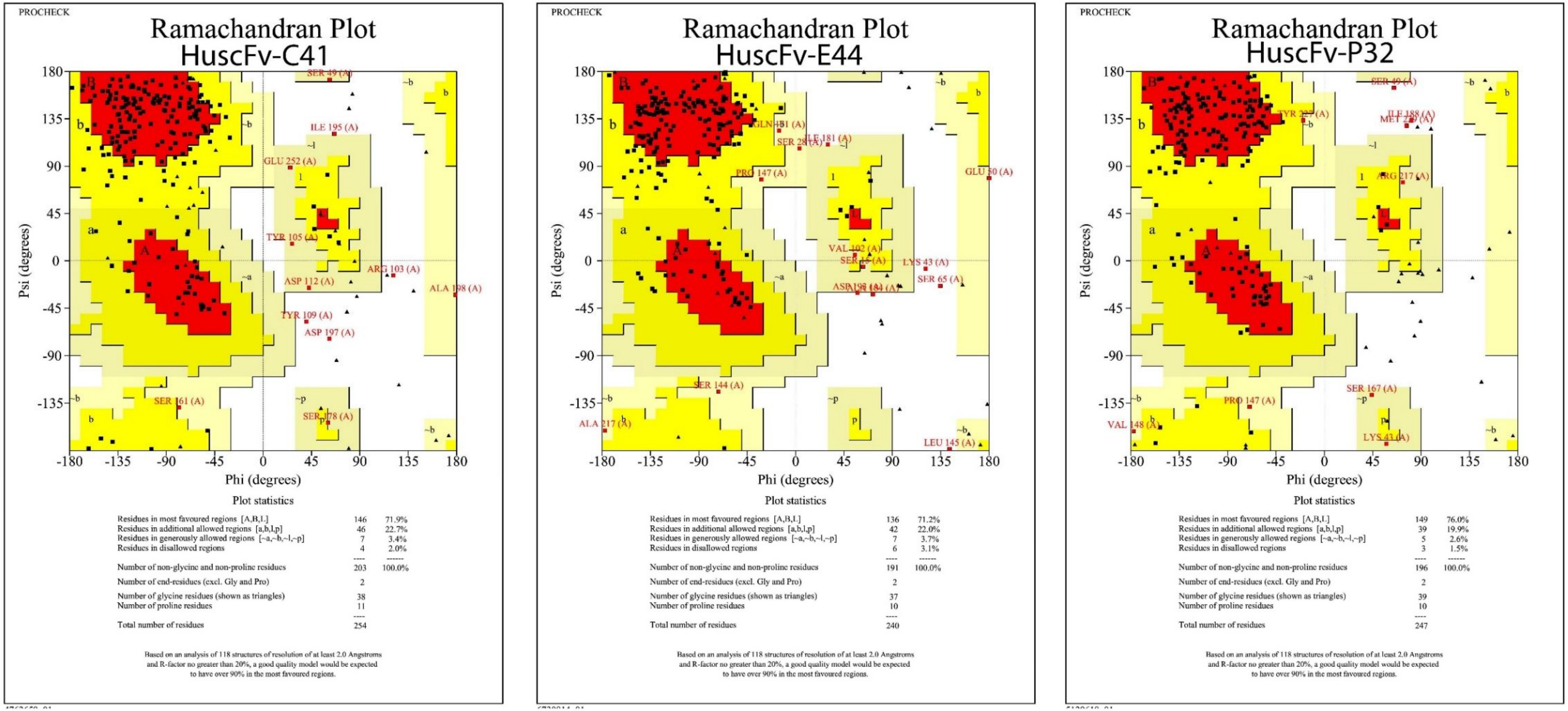

Supplementary Figure S4. Ramachandran diagrams of the modeled HuscFv-C41, HuscFv-E44, and HuscFv-P32.

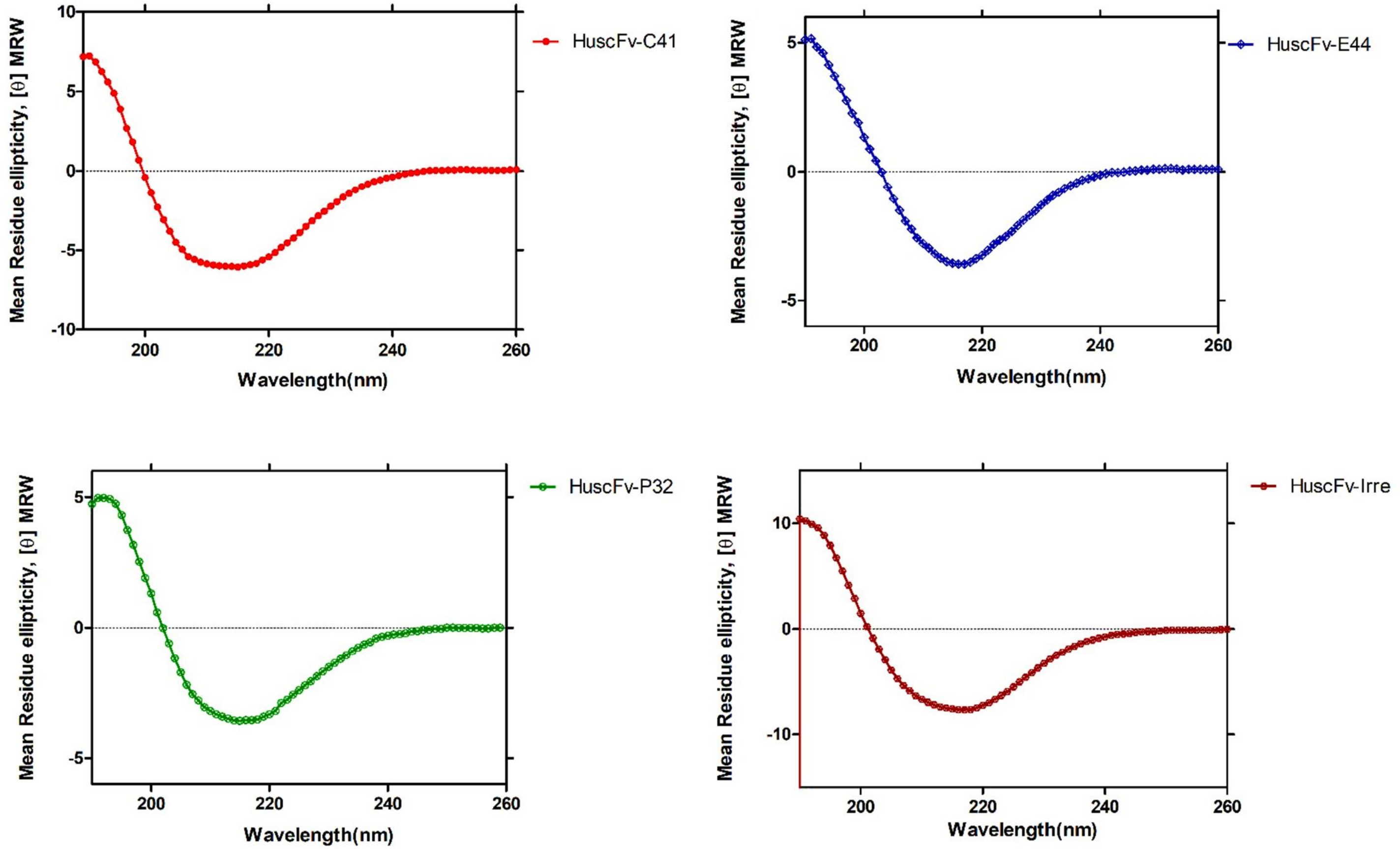

Supplementary Figure S5. CD spectra from Far-UV Circular Dichroism (CD) measurements of the refolded recombinant HuscFv-C41, HuscFv-E44, HuscFv-P32, and HuscFv-Irre (control HuscFv).

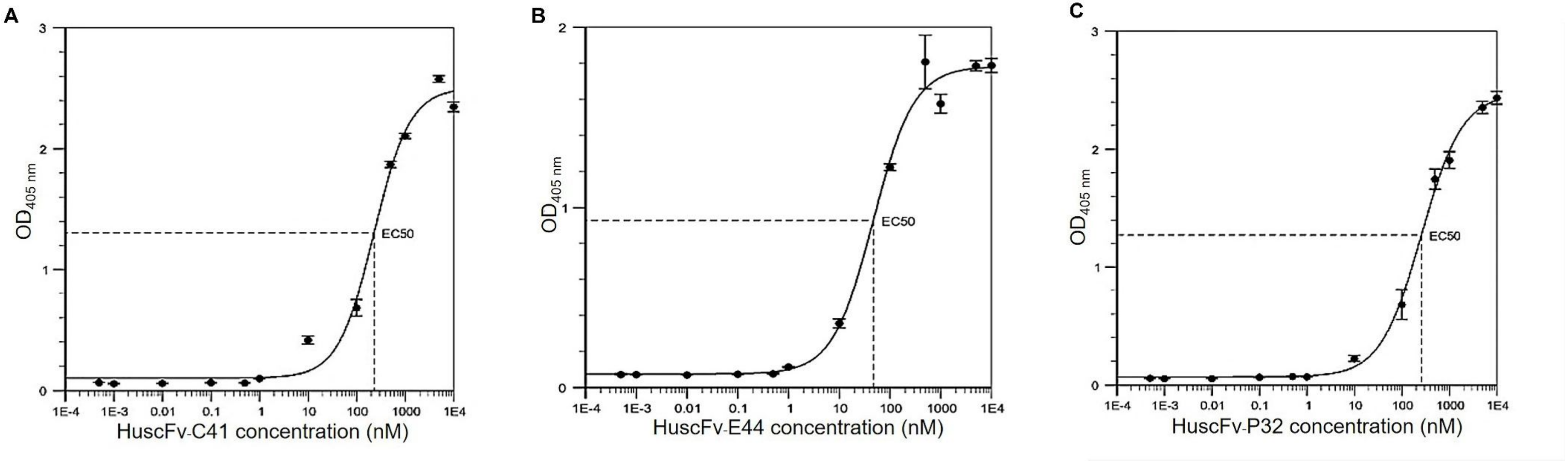

Supplementary Figure S6. EC$_{50}$ of refolded recombinant HuscFvs that bound to ETA as determined by indirect ELISA. Serial concentrations of purified, refolded HuscFvs at 0.5 pM to 10 μM were incubated with immobilized ETA (50 nM) in EIA/RIA wells. OD$_{405nm}$ signals were determined against blank (antigen-coated well incubated with diluent). The EC$_{50}$ graphs of (A) HuscFv-C41, (B) HuscFv-E44, and (C) HuscFv-P32, were generated using EC$_{50}$ Calculator (https://www.aatbio.com/tools/ec50-calculator).
